# *In silico* identification of potential inhibitors of Mycobacterium tuberculosis MmpS5L5 from the ReFRAME database: a structure-based virtual screening, molecular docking and molecular dynamics approach

**DOI:** 10.3389/fcimb.2026.1812089

**Published:** 2026-04-24

**Authors:** Caroline Maina, Edwin Murungi, Elizabeth Kigondu

**Affiliations:** 1 Department of Chemistry, University of Cape Town, Cape Town, South Africa; 2 Department of Medical Biochemistry, Kisii University, Kisii, Kenya; 3 Centre for Traditional Medicine and Drug Research, Kenya Medical Research Institute, Nairobi, Kenya

**Keywords:** drug efflux, drug repurposing, MMGBSA binding free energy, molecular docking & molecular dynamics (MD) simulation, *Mtb* (*Mycobacterium tuberculosis*), principal component analyses (PCA), virtual screening, structure-based drug design

## Abstract

**Introduction:**

Tuberculosis (TB), caused by *Mycobacterium tuberculosis* (*Mtb*), is the leading infectious cause of death globally, disproportionately impacting low- and medium-income countries (LMICs). The emergence and transmission of drug resistant *Mtb* strains has rendered a majority of the current anti-TB agents ineffective and significantly complicated TB treatment. Thus, the development of new anti-TB remedies with novel modes of action is a pressing priority. An attractive, viable strategy is the development of potentiators of anti-TB drugs that reverse drug efflux, a key intrinsic *Mtb* drug resistance mechanism. Targeting *Mtb* MmpS5L5, a critical efflux pump (EP) implicated in the mycobacterial expulsion of various anti-TB drugs including bedaquiline, tetracyclines, azoles and clofazimine would likely enhance the efficacy of current anti-TB drugs by preventing the development of drug resistance.

**Methods:**

The recent determination of a high-resolution crystal structure of *Mtb* MmpS5L5 (PDBID: 8ZKP) enables the utilisation of structure-anchored approaches for the uncovering of probable efflux inhibitors. In this study, pharmacophore models developed using the *Mtb* MmpS5L5 three-dimensional (3-D) structure and its known inhibitors, verapamil and norverapamil, were thereafter utilised for the screening of the REFRAME database, a comprehensive drug repurposing library, to identify novel ligand scaffolds with putative activity against the EP. Predicted target binding affinity for the top candidates was ascertained and validated using molecular docking and 100 ns molecular dynamics (MD) simulations, respectively. Further, post-MD analysis including Molecular Mechanics/Generalized Born Surface Area calculations (MMGBSA), Principal Component Analysis, and Free Energy Landscapes were done to study thermodynamic and conformational dynamics of the complexes.

**Results:**

Six compounds (406, 3920, 4031, 4787, 7104, 10367) had stronger predicted binding affinities for MmpS5L5 than the known inhibitors, with docking scores ranging from -8.70 to -5.01 kcal/mol and had predicted protein contacts similar to those of the validated inhibitors. Molecular dynamic simulations and MMGBSA analyses demonstrated stable and energetically favourable protein-ligand interaction. Among the six compounds, 3920 and 4031 emerged as the most promising hits as their average total ΔG bind (-111.81 ± 8.98 kcal/mol and -109.56 ± 8.40 kcal/mol respectively) and ligand efficiency (-16.46 ± 4.06 kcal/mol and -17.63 ± 1.27 kcal/mol) were lower than those of the reference inhibitors.

**Discussion:**

This study identified compounds from the ReFRAME database that may provide putative scaffolds for the development of *Mtb* efflux inhibitors that can potentiate the treatment efficacy of current anti-TB drugs. Further *in vitro* and *in vivo* studies are needed to validate their inhibition potential.

## 1 Introduction

Tuberculosis (TB), caused by *Mycobacterium tuberculosis* (*Mtb*), is the leading infectious killer from a single pathogenic agent worldwide. Of the estimated 1.23 million deaths and 10.7 million new TB cases reported in 2024, 88% of the fatalities and new infections occurred in Western Pacific, Africa, and South-East Asia (World Health Organization, 2025). The current WHO recommended TB treatment regimen is lengthy (minimum 4 months) and is prone to noncompliance which amplifies the risk of the development of drug resistance. The first-line agents in the current regimen, namely rifampicin, pyrazinamide, ethambutol and isoniazid are usually used in combination with moxifloxacin, linezolid, clofazimine (CFZ) and bedaquiline (BDQ) as second-line agents. However, the efficacy of TB treatment regimens is significantly hampered by the increasing prevalence of multidrug resistant (MDR) and extensively drug resistant (XDR) *Mtb* strains (Graciaa et al., 2023; Saukkonen et al., 2025). Thus, there is a pressing imperative to develop new anti-TB drugs with novel mechanisms of action that can be combined with the current drugs to augment their efficacy.

An emerging, viable strategy for potentiating the efficacy of current anti-TB drugs entails inhibiting drug efflux, a potent mechanism deployed by *Mtb* to counter drug action (Ghajavand et al., 2019; Laws et al., 2022). Indeed, increasing reports of drug efflux conferring drug resistance to an array of *Mtb* clinical isolates has highlighted the need to target *Mtb* efflux pumps (EPs) (Rodrigues et al., 2020). For instance, the up-regulation of the MmpS5L5 EP due to mutations in its transcriptional repressor Rv0678 has been implicated in the extrusion of various anti-TB drugs including CFZ, tetracyclines, azoles, and the recently approved agent for XDR and MDR TB infections, BDQ (Chacha et al., 2025; Hu et al., 2025; Madadi-Goli et al., 2024). It has recently been demonstrated that CRISPRi knock down of MmpS5L5, as well as inhibition of MmpS5L5 by verapamil (VER) and norverapamil (NOR), restored CFZ and BDQ susceptibility in resistant *Mtb* strains to the wild type level, highlighting MmpS5L5 inhibitors as probable potentiators of current anti-TB drugs (Fountain et al., 2025a, 2025b).

MmpS5L5 is an RND-family efflux system in which MmpL5 functions as the large membrane transporter and MmpS5 serves as its associated accessory protein (Fountain et al., 2025a). MmpL5 is a multipass inner-membrane protein with 12 transmembrane helices and extensive periplasmic/extracellular domains, while MmpS5 is a single transmembrane protein with a C-terminal immunoglobin-like domain that contributes to proper assembly and stabilization of the functional transporter complex (Fountain et al., 2025a; Xiong et al., 2025; Yamamoto et al., 2021). Structural and functional studies established that disrupting the MmpS5-MmpL5 interface and inhibiting MmpL5 abolishes the pump’s efflux activity, reducing the minimum inhibitory concentration of its substrates, including BDQ and CFZ (Fountain et al., 2025a; Xiong et al., 2025; Yamamoto et al., 2021). In addition to its role in drug efflux, the MmpS5L5 system is involved in siderophore export, contributing to iron acquisition and mycobacterial physiology. This dual role in both drug resistance and fitness further supports targeting the MmpS5L5 system.

An attractive computational strategy routinely utilised to mainstream drug discovery efforts is the structure-based drug discovery (SBDD) (Ejalonibu et al., 2021). Specifically, this approach enables the delineation of candidate compounds whose molecular structures match the characteristics of the receptor binding site, and thus potentially bind with stronger affinities. Indeed, SBDD has successfully been used in anti-TB drug discovery endeavours (Bruch et al., 2020; Kingdon and Alderwick, 2021). Although computational methods have been utilised in the uncovering of promising lead compounds that could further be developed as MDR- and XDR-TB drugs (Biswas et al., 2021), the use of SBDD to guide the discovery of *Mtb* efflux inhibitors has been undermined by the sparse structural information on *Mtb* EPs because they do not readily crystalize (Bruch et al., 2020; Zhang et al., 2019). SBDD approaches usually use crystal structures of proteins because they have been validated experimentally, thus they are more accurate than homology models (Karelina et al., 2023; Kingdon and Alderwick, 2021). Thus, the recent experimental determination of a high-resolution cryo-electron microscopy (Cryo-EM) structure of Mtb MmpS5L5 (Protein Data Bank (PDB) ID: 8ZKP) has enabled the use of SBDD in the unveiling of potential MmpS5L5 inhibitors that could be developed as augmenters of current anti-TB drugs. In this study, we have utilised SBDD approaches to uncover compounds from the ReFRAME (Repurposing, Focused Rescue, and Accelerated Medchem) database, a comprehensive drug repurposing library, that can be further investigated as potential *Mtb* MmpS5L5 inhibitors.

## 2 Methods

The computational work described herein was performed using GROMACS (2024.2) and the Maestro graphical user interface (GUI) within the Schrodinger Suite software package (Schrödinger, LLC, NY, 2024-3) (Abraham et al., 2015; Baltrukevich and Podlewska, 2022; Shirts et al., 2017). Specifically, the Maestro GUI was utilized for protein and ligand preparation, pharmacophore-based virtual screening, molecular docking, and molecular dynamics (MD) simulations while GROMACS was used for post-MD analyses.

### 2.1 Protein preparation, binding site identification, and pharmacophore modelling

The atomic structure of the *Mtb* MmpS5L5 complex (PDB ID: 8ZKP), was retrieved from the PDB, imported into Maestro, and prepared using the Protein Preparation tool. Briefly, missing loops and side chains were predicted using the Prime tool while the hetero states were generated using the Epik application. Moreover, preceding structure minimisation using the OPLS4 force field, hydrogen bond assignments were optimized using PROPKA (Kurki et al., 2024). Finally, water molecules within 5 Å radius of heteroatoms were deleted. Prediction and assessment of the druggability of putative binding sites was inferred using SiteMap (Ge et al., 2024). Only binding sites with a druggability score of at least 0.8 were selected for receptor grid generation. These binding sites were subjected to molecular docking and molecular dynamics (MD) simulations with known direct inhibitors of *Mtb* MmpS5L5, namely VER and NOR (Fountain et al., 2025a; Xu et al., 2017), to validate them as probable binding sites in MmpS5L5. Following validation, pharmacophore models were generated using the Phase module.

### 2.2 Pharmacophore-based virtual screening

The SMILES notations of the compounds in the ReFRAME database were obtained and imported into Maestro (Janes et al., 2018). Following removal of duplicate ligands, the Phase module was used to create a screening database of the compounds (Moussa et al., 2021). For each compound, a maximum of 50 3D conformers were generated and minimized using the OPLS4 force field. Possible states were generated at a pH of 7.0 ± 2.0 and chiralities determined from the 3D structures using Epik. The prepared ligands were then filtered to remove those with reactive functional groups. The chosen pharmacophore models were then utilised to screen the ReFRAME Phase database to uncover compounds with full matches to the created pharmacophore models that could be explored as potential hits.

### 2.3 Molecular docking

The 3D structures of the hits obtained from virtual screening were docked onto the receptor grids of the selected binding sites using the Glide Ligand Docking tool in extra precision (XP) mode which undertakes extensive sampling to minimise false positives (Friesner et al., 2006). The compounds were ranked based on their docking scores, and the three highest-ranked compounds with scores lower than those of the reference inhibitors were selected as hits and subjected to MD simulations.

### 2.4 Molecular dynamics simulations

To assess conformational stability, 100-ns MD simulations of *Mtb* MmpS5L5 bound to the hits from the docking step were performed using DESMOND, while GROMACS was used to analyse the subsequent MD trajectories (Farhadi et al., 2025). Since MmpS5L5 is a transmembrane protein, the System Builder tool was used to build a POPC (1-palmitoyl-2-oleoyl-sn-glycero-3-phosphocholine) membrane around its helixes as described in UniProtKB: P9WJV0 (The UniProt Consortium, 2025). A solvated system within an orthorhombic periodic boundary box, with dimensions uniformly set to 10 Å for each axis, and the angles fixed at 90° was built using the TIP3P system. To maintain charge neutrality, chloride ions (Cl-) were added into the system based on the model’s overall charge. Furthermore, a 0.15 M salt concentration was set to mimic the physiological conditions of body fluids before the model was minimised. The minimised model was parameterized using the OPLS4 force field and relaxed using the default Desmond NPT relaxation protocol prior to production simulation. The 100-ns production run was performed in the NPT ensemble at 300 K and 1.01 bar using the Nosé–Hoover chain thermostat and the Martyna–Tobias–Klein barostat respectively. The RESPA integrator was applied with bonded, near, and far time steps of 2.0, 2.0, and 6.0 fs, respectively, and a 9.0 Å cutoff was used for short-range non-bonded interactions. A comprehensive structure analysis of the MD trajectory was conducted using GROMACS.

### 2.5 Molecular mechanics/generalized born surface area calculations

To compute the binding free energy of multiple snapshots of the MD trajectory to ascertain the thermodynamic stability of the complexes during the MD simulations, the thermal_mmgbsa.py script of the Prime module in the Schrödinger Suite was used (Genheden and Ryde, 2015). For these calculations, the frames for the entire 100 ns of the MD trajectory file were utilised. Individual energy modules including Coulombic, solvation, van der Waals, lipophilic, covalent, hydrogen bond, and electrostatic energies of the protein, complex, and ligand were used to compute the MMGBSA binding free energy in kcal/mol (Bharadwaj et al., 2024). The total binding free energy was calculated using the following formula:


$$\Delta G_{bind}=G_{complex}-(G_{protein}+G_{ligand})$$

Where,

$\Delta G_{bind}$: binding free energy; G_complex_: complex’s free energy; G_protein_: target protein’s free energy; and, G_ligand_: ligand’s free energy.

### 2.6 Principal component analysis-based free energy landscape analysis

Principal Component Analysis (PCA) is a multivariate statistical approach that uses eigenvectors and eigenvalues to delineate the dominant motions of protein-ligand complexes during MD simulations (Sethi et al., 2026). Briefly, the simulation convergence was estimated from the cosine values for the dominant eigenvectors identified after diagonalising the covariance matrix of the protein backbone using the entire 100-ns of the MD trajectories (Amadei et al., 1993). The free energy landscape (FEL) analysis was conducted to visualise the energy of the sampled conformational substates, allowing inference of into the thermodynamic stability of receptor-ligand complexes (Shahbaaz et al., 2022). Three-dimensional PCA-FEL diagrams were constructed using the *gmx sham* module and the first two principal components (PC1 and PC2) identified using the *gmx eigen* module in GROMACs. The plots generated were visualised in Matplotlib.

## 3 Results and discussion

**Figure 1:**
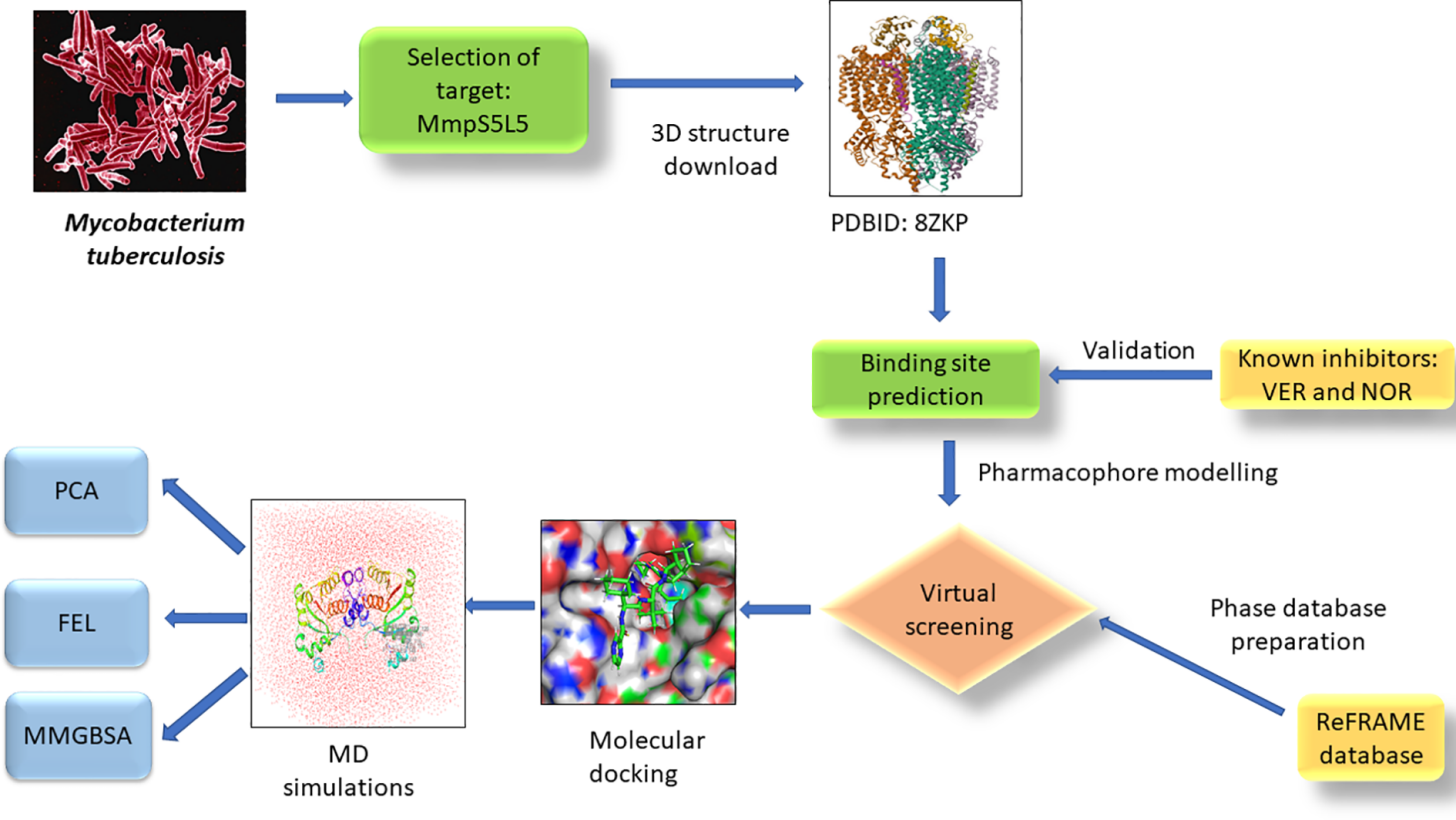
Schema of the SBDD strategy used in the identification of putative Mtb MmpS5L5 inhibitors.

The study’s computational workflow is depicted in **Figure 1**.

### 3.1 Binding site identification and validation

**Figure 2:**
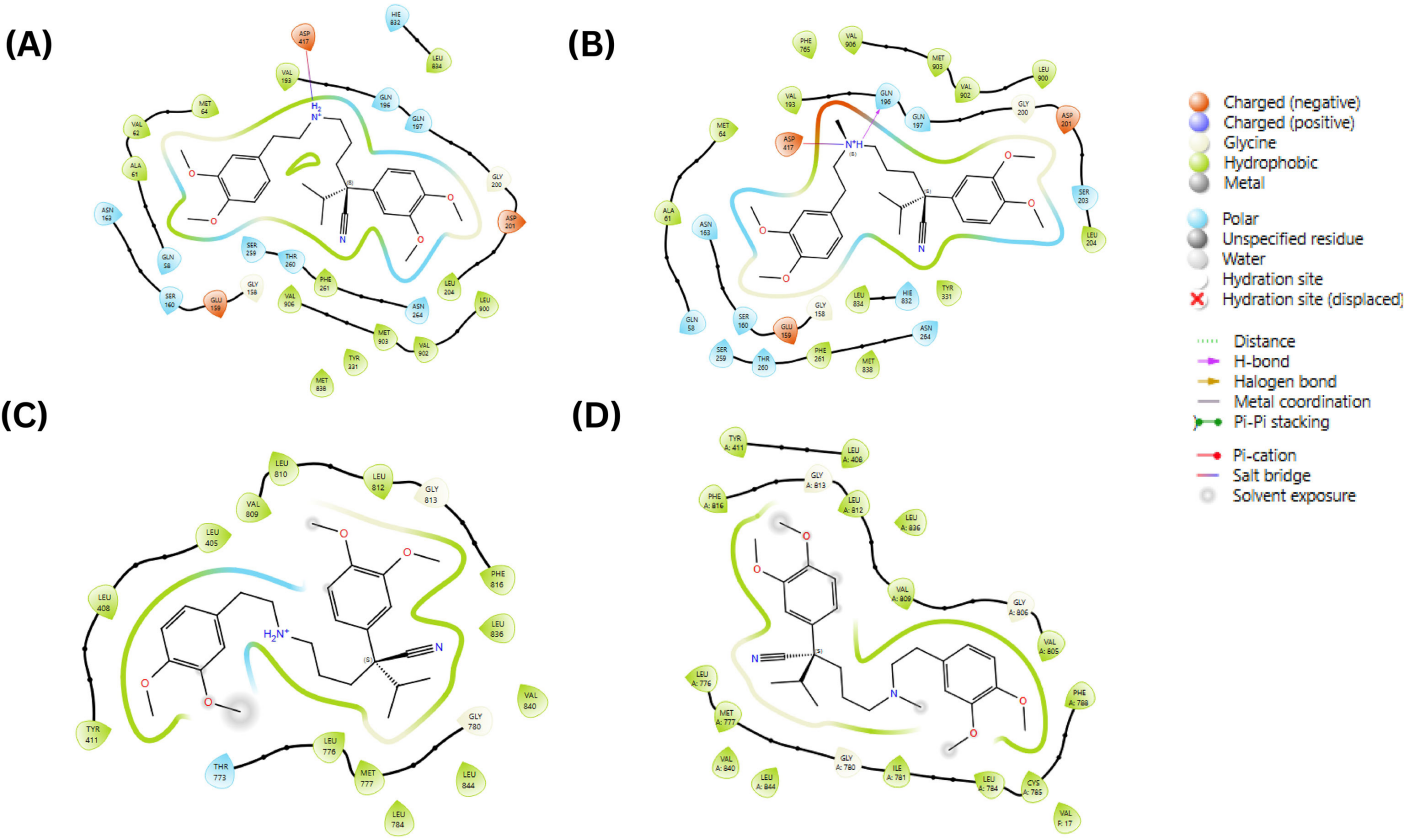
Two-dimensional (2D) representation of key protein contacts made by NOR in Site 1 **(A)**, VER in Site 1 **(B)**, NOR in Site 4 **(C)** and VER in Site 4 **(D)**.

**Figure 3:**
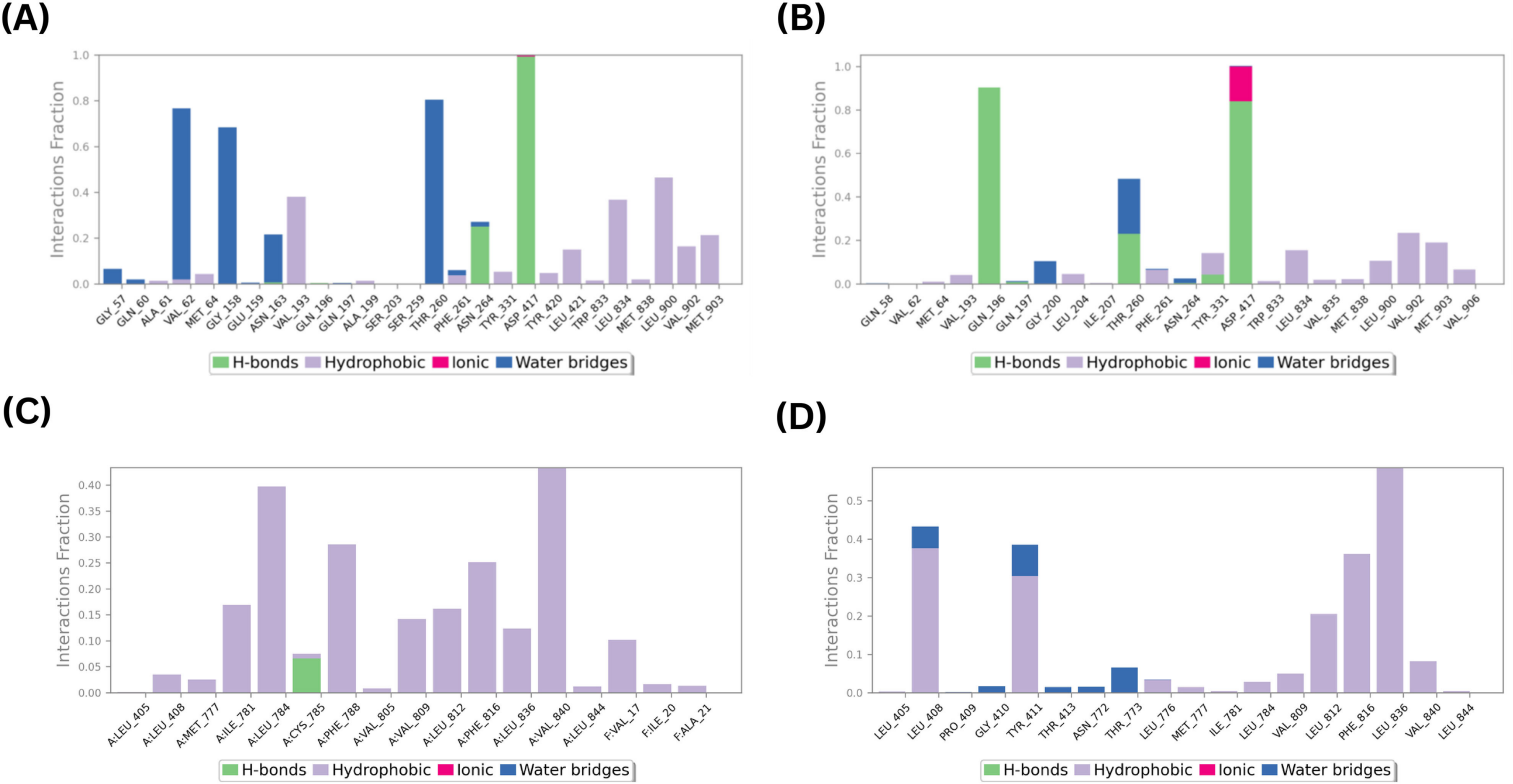
Key MmpS5L5 amino acid residue contacts for VER and NOR in Site 1 (**A, B** respectively) and Site 4 (**C, D**, respectively).

The SiteMap tool predicted four binding sites (1-4) with site and druggability scores of at least 0.8 in the apo structure of MmpS5L5 (PDB ID: 8ZKP) (Xiong et al., 2025). Docking of the known direct inhibitors of MmpS5L5 (VER and NOR onto the four sites revealed favourable binding only onto sites 1 and 4 as shown in **Figure 2**. Site 1 maps onto the substrate-binding cavity within MmpL5’s periplasmic domain while Site 4 maps onto the MmpS5-MmpL5 interface (Fountain et al., 2025a; Xiong et al., 2025). The predicted binding scores for NOR and VER onto site 1 were -2.60 and -3.81 kcal/mol respectively as depicted in **Figures 2A, C** respectively. On the other hand, as shown in **Figures 2B, D**, the docking scores for NOR and VER onto site 4 were -4.66 and -3.95 kcal/mol respectively. Given the relatively weak ligand binding illustrated by the docking scores of > -5.0 kcal/mol, the top ranked inhibitor docked poses in the two sites were subjected to MD simulations to determine the stability of the molecular docking predicted ligand binding and validate the predicted binding sites (Breznik et al., 2023). Ligand interactions analysis depicted in **Figure 3** revealed that the reference inhibitors NOR and VER made stable contacts with several key MmpS5L5 residues within each binding pocket, validating the sites as putative binding sites.

Specifically, as shown in **Figure 3A**, VER in site 1 formed stable water bridges with VAL-62, GLY-158, ASN-163, and SER-259; hydrophobic contacts with VAL-193, LEU-834, LEU-900, VAL-902, and MET-903; and hydrogen bonds with ASN-264 and ASP-417. On the other hand, as illustrated in **Figure 3B**, NOR formed stable water bridges with THR-260; hydrophobic contacts with VAL-902 and VAL-903; and hydrogen bonds with GLN-196, THR-260 and ASP-417. Functional and structural analyses have revealed GLN-196, ASP-417 and VAL-902 as critical residues in the efflux activity of *Mtb* MmpS5L5, with the mutation of these residues demonstrated to significantly alter the minimum inhibitory concentrations (MICs) of the pump substrates including BDQ and CFZ (Fountain et al., 2025a). The formation of stable interactions between VER and NOR and these residues points to the probability of site 1 being a binding site for both inhibitors and thus an e-pharmacophore model was developed for the site.

As illustrated in **Figure 3C**, in site 4, VER formed persistent hydrogen bonds with CYS-785, and hydrophobic interactions with ILE-781, LEU-784, PHE-788, PHE-816, VAL-840 of MmpL5 and VAL-17 of MmpS5 while NOR formed stable hydrophobic contacts with LEU-408, TYR-411, LEU-812, PHE-816, LEU-836, and VAL-840 of MmpL5 as shown in **Figure 3D**. It has been established that disrupting the MmpS5-MmpL5 interface abolishes the pump’s efflux activity (Fountain et al., 2025a; Xiong et al., 2025; Yamamoto et al., 2021). Thus, the inhibitors interacting with MET-777, ILE-781, CYS-785, and PHE-788 of MmpL5 and VAL-17 and ALA21 of MmpS5 may plausibly disrupt the extensive hydrophobic interactions between the MmpS5 membrane region and MmpL5’s transmembrane helix 8. The fact these interactions are vital stabilizers of the MmpS5L5 complex points to the probability of site 4 being a likely binding site and therefore an e-pharmacophore model was developed for this site.

### 3.2 Virtual pharmacophore screening and molecular docking

Screening of the ReFRAME database using the e-pharmacophore models generated for sites 1 and 4 revealed 21 and 30 potential hits for the two sites respectively that matched at least 5 of their pharmacophoric features. Docking of the virtual screening hits onto the two sites was performed using GLIDE in the XP mode. Of the 21 compounds docked onto MmpS5L5 site 1, 16 had lower docking scores compared to the VER and NOR. Further analysis of their poses and key protein interactions revealed that 6 compounds made similar interactions and adopted similar poses to those of the reference inhibitors. On the other hand, of the 30 compounds docked onto MmpS5L5 site 4, only 9 had lower docking scores than NOR and VER. Probing of their poses and key protein interactions demonstrated that 7 compounds had hydrophobic contacts and poses akin to those of the reference inhibitors. The 2-D structures and key receptor interactions of the three top-ranked compounds for each binding site based on docking scores are summarized in **Table 1**.

**Table 1:** Docking scores, structures and types of key interactions of the 3 top-ranked compounds with amino acids in the predicted Mtb MmpS5L5 binding sites.

Site 1			
Compound ReFRAME Code	2D structure	Docking score (kcal/mol)	Types of key protein interactions
4787	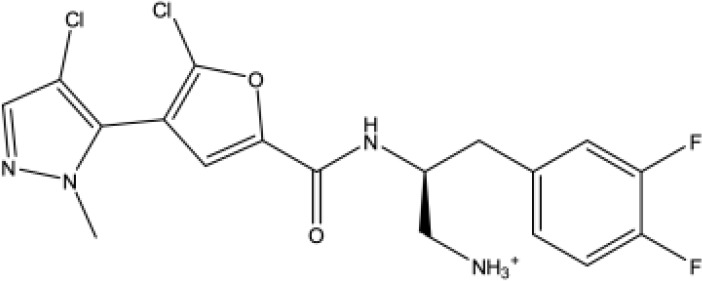	-8.70	Salt bridge: **ASP-417**
3920	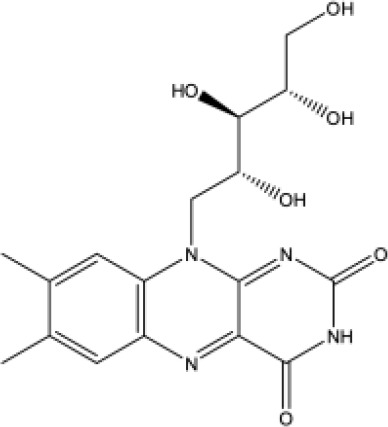	-8.11	H-bond: GLY-158, **GLN-196**, ASP-201, ASN-264
406	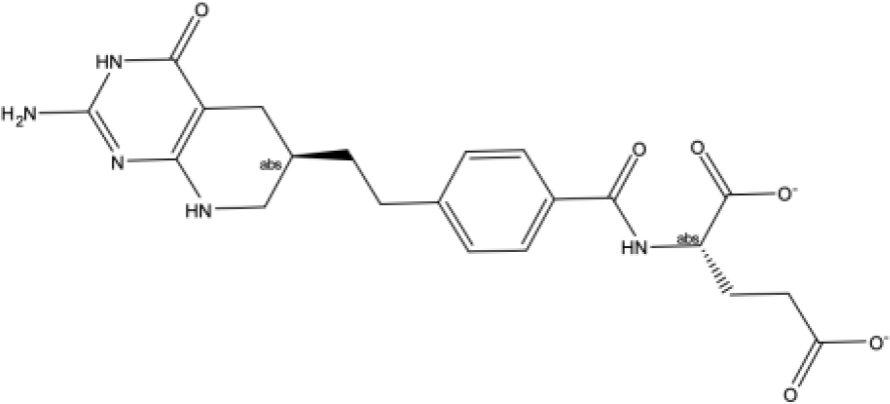	-6.96	H-bond: GLY-158; salt bridge: **ASP-417**
Site 4			
Compound REFRAME Code	2D Structure	Docking score (kcal/mol)	Types of key protein interactions
4031	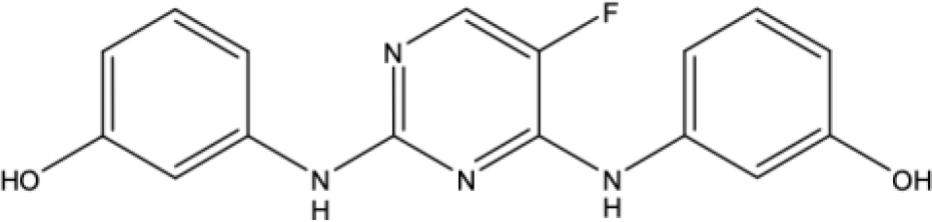	-5.19	H-bond: **LEU 776**, THR-773
7104	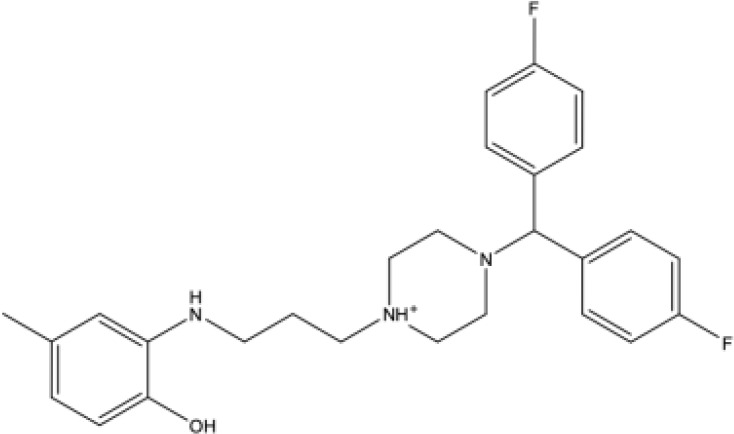	-5.02	H-bond: THR-773
10367	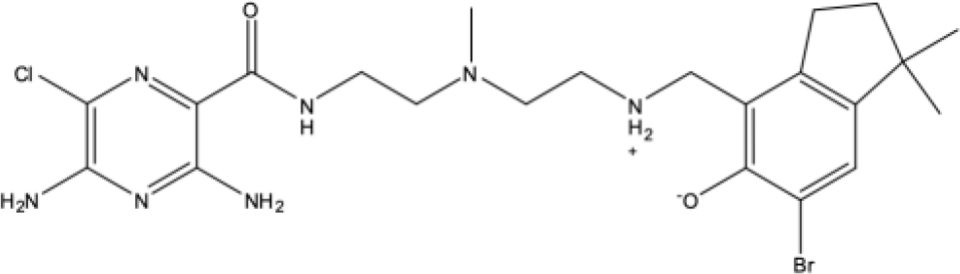	-5.19	H-bond: **CYS-785**

Highlighted in bold are MmpS5L5's amino acids established to be crucial to its efflux function.

### 3.3 MD-based stability analysis

**Figure 4:**
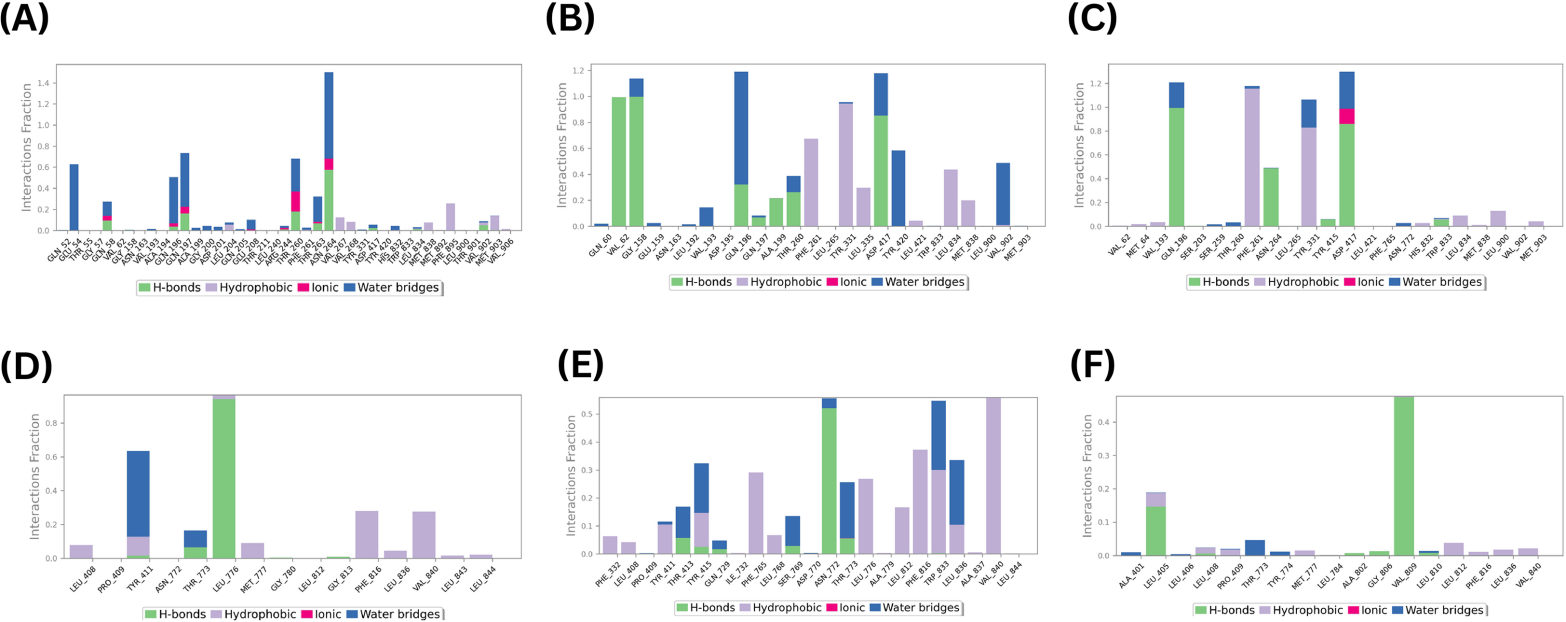
Key amino acid residue contacts formed by potential hits 406 **(A)**, 3920 **(B)**, and 4787 **(C)** in Site 1, and 3710 **(D)**, 7104 **(E)**, and 10367 **(F)** in Site 4 of *Mtb* MmpS5L5.

To assess the stability of the predicted protein-ligand contacts, the docked complexes for the top 3 ranked compounds for each binding site were subjected to 100 ns MD simulations. A time-course analyses of the interactions between the hits and MmpS5L5 sites 1 and 4 amino acid residues are shown in **Figure 4**.

All of the putative hits in site 1 formed stable contacts with several amino acid residues essential for MmpS5L5 efflux activity, as depicted in **Figure 4A** (406), 4B (3920), and 4C (4787). Markedly, all three hits formed stable interactions with GLN-196 which has been implicated in substrate recognition (Fountain et al., 2025a). In addition, 3920 established stable interactions with TYR-331, ASP-417, and VAL-902 while 4787 made stable contacts with TYR-331 and ASP-417. Since the ligand interaction analyses illustrated that the hits formed stable interactions with amino acid residues pivotal for the efflux function of MmpS5L5, the root mean square fluctuation of the protein residues (PMRSF), the root mean square deviation (RMSD) of the protein’s alpha carbons (PRMSD) and ligand with respect to the protein backbone (LRMSD), radius of gyration (Rg), and solvent accessible surface area (SASA) were computed to assess the structural stability of the systems (Kordylewski et al., 2025).

In contrast, none of the hits in site 4 displayed stable contacts with any of the amino acid residues involved in the stabilisation of the MmpL5-MmpS5 interface (Xiong et al., 2025), as shown in **Figure 4D** (4031), 4E (7104), and 4F (10367). Nonetheless, their occupancy of Site 4 could still destabilise the interface. Thus, the PRMSD, LRMSD, PRMSF, Rg, and SASA of the systems were also calculated and compared to those of the reference inhibitors to determine their potential to inhibit MmpS5L5 efflux function.

**Figure 5:**
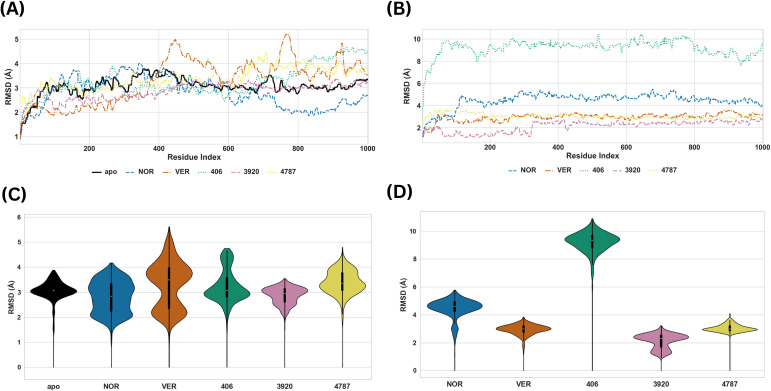
Time-series and violin plots of the PRMSD (**A, C** respectively) and LRMSD of the MmpS5L5-inhibitor and -hit systems (**B, D** respectively) at Site 1.

The PRMSD and LRMSD analyses were first examined to determine the global stability of the protein backbone and the persistence of ligand binding within each site. The PRMSD provides information regarding variation of the protein’s backbone structure during MD simulations and whether the system had equilibrated at the conclusion of the simulation, thus indicating the global stability of the complex (Manandhar et al., 2022). On the other hand, LRMSD assesses the ligand’s stability within the binding pocket, which is critical because a drug’s residency time within a receptor influences its efficacy (Nada et al., 2022; Reading et al., 2020). The time-series and violin plots of the PRMSD of the MmpS5L5-inhibitor and -hit systems at Site 1 are illustrated in **Figures 5A, C** whereas their LRMSD are provided in **Figures 5B, D** respectively.

The apo protein exhibited a moderate PRMSD range with a compact unimodal violin plot, consistent with the flexible structure of MmpS5L5 (Fountain et al., 2025a). The PRMSD of the complex with VER bound at site 1 was greater than 4 Å after 40 ns, causing its violin plot to be bimodal and implying that VER binding caused rearrangements in MmpS5L5’s global structure. The LRMSD was consistently around 3 Å, suggesting it was stable within the site despite the broader PRMSD. In contrast, the PRMSD of the NOR complex was lower than that of the apo protein after 40 ns, indicating restraint of the global structure. Its LRMSD rose rapidly to > 4 Å and stabilised after about 10 ns, indicating that NOR initially repositioned then maintained its pose.

Among the Site 1 hits, the 406 and 4787 complexes maintained relatively low PRMSD values early in the simulation, although both increased after about 60 ns, indicating moderate destabilization of the global structure. Conversely, the PRMSD of the 3920 complex traced that of the apo after 40 ns, indicating that ligand binding stabilised MmpS5L5’s structure. 3920 and 4787 displayed low and compact LRMSD distribution, indicating tight and stable binding. In contrast, the LRMSD of 406 rose rapidly to about 10 Å then stabilised, indicating substantial initial ligand orientation in the binding pocket. Its time-series plot was relatively rugged, suggesting that it kept readjusting inside the binding pocket. Taken together, these findings point to 3920 as the most stable Site 1 hit, with 4787 also showing favourable binding behaviour, while 406 appeared comparatively more mobile.

**Figure 6:**
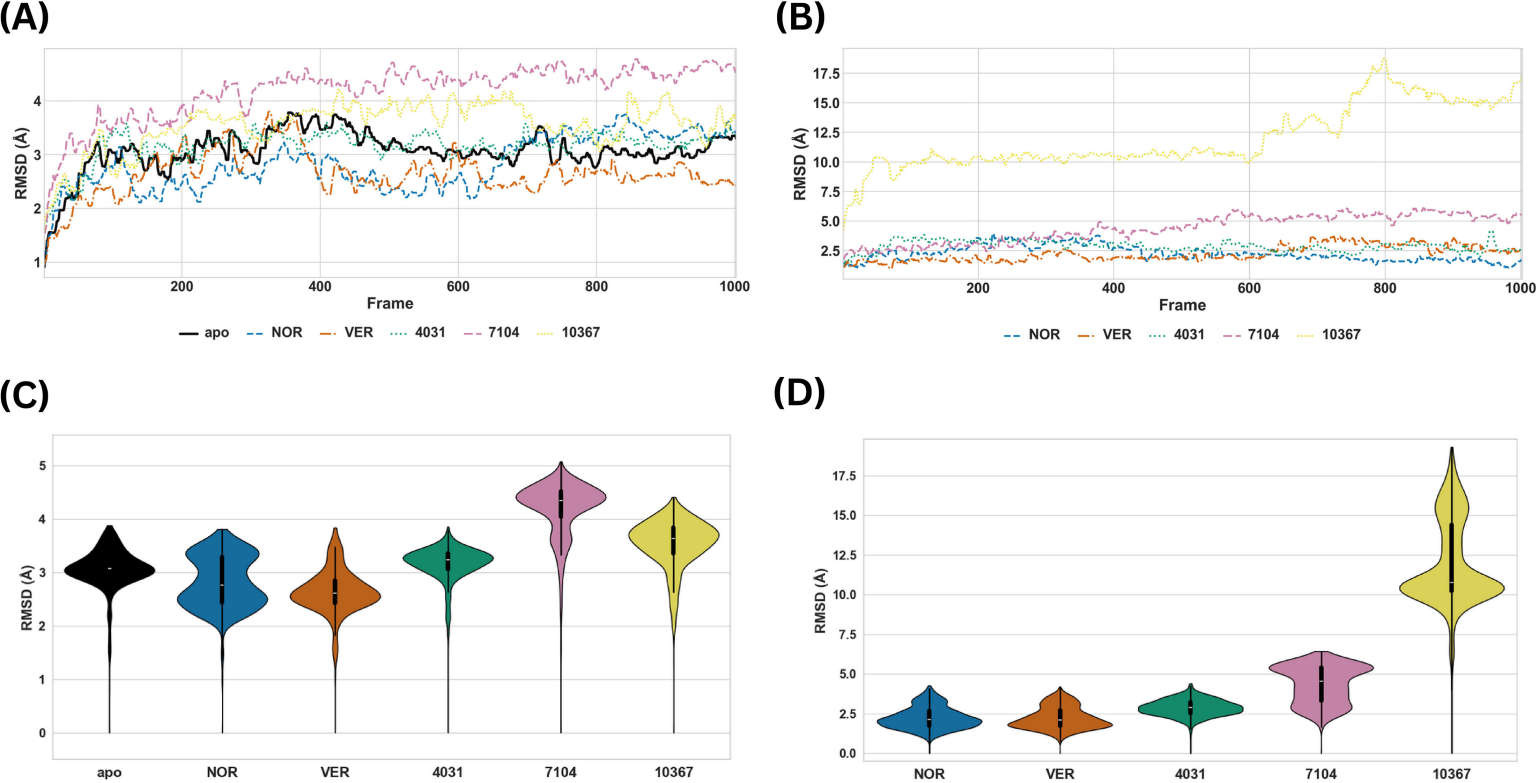
Time-series and violin plots of the PRMSD (**A, C** respectively) and LRMSD of the MmpS5L5-inhibitor and -hit systems (**B, D** respectively) at Site 4.

A similar analysis was carried out for the Site 4 systems. The violin and time-series plots of the PRMSD of the MmpS5L5-inhibitor and -hit systems at Site 4 are depicted in **Figures 6A, C** while plots of their LRMSD are shown in **Figures 6B, D**.

The PRMSD of the VER complex was consistently lower than that of the apo protein after 30 ns, indicating that VER binding constrained MmpS5L5’s global structure. The PRMSD of the NOR complex mirrored that of the VER complex until after 60 ns, where it rose slightly above the apo one, consistent with significant structural rearrangement. The LRMSDs of NOR and VER were consistently low throughout the simulation, suggesting they were stable within the binding pocket.

Among the Site 4 hits, the 4031 complex maintained a PRMSD profile close to those of the apo and the reference inhibitors, indicating that the protein core structure was unaffected by ligand binding. Its LRMSD was low, indicating a stable binding pose after a slight initial adjustment. In contrast, 10367 and 7104 complexes had a consistently higher PRMSD than the apo throughout the simulation, suggesting destabilization of MmpS5L5’s global structure. The LRMSD of 7104 was low for the first 30 ns followed by a modest increase to >5 Å after 40 ns, implying a mobile binding mode. The LRMSD of 10367 was very high (~10–18 Å), suggesting that the ligand was unstable in the binding pocket. Overall, these profiles revealed 4031 as the most stable Site 4 hit.

**Figure 7:**
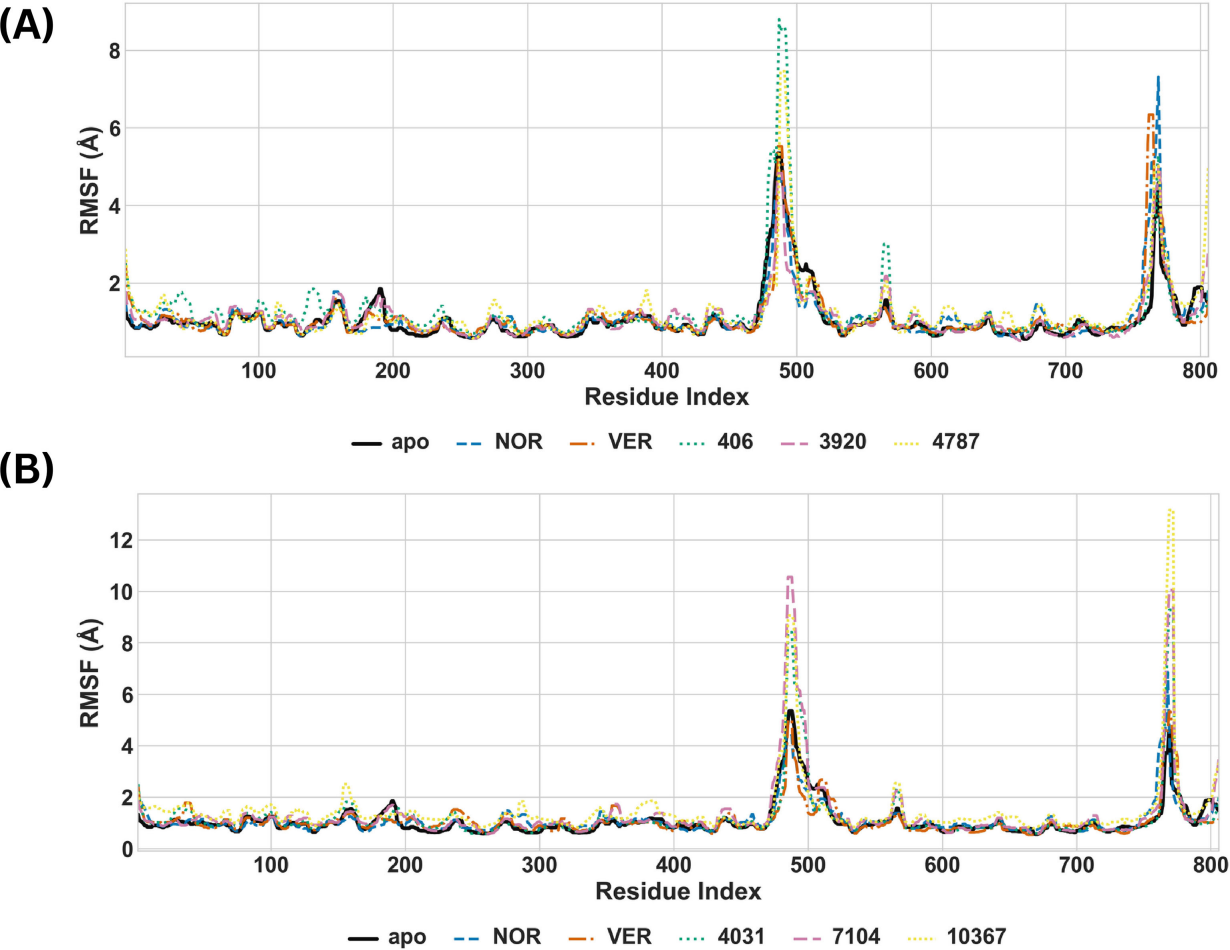
Time-series plots of the PRMSF values for the MmpS5L5-apo, -inhibitor, and -hit complexes at Site 1 **(A)** and Site 4 **(B)**.

Next, the PRMSF values for the apo and ligand-bound systems were calculated to assess the impact of ligand binding on flexibility of MmpS5L5’s amino acid residues. The plots of the PRMSF values during simulations of the complexes at Sites 1 and 4 are depicted in **Figures 7A, B** respectively.

The PRMSF values of the apo system indicated that the core of MmpS5L5 was stable, with two major flexibility hotspots – Peak 1 (LYS-490 – SER502 and MET-669 – LEU-705 of MmpL5) and Peak 2 (GLN-945 – ALA-956 of MmpL5 and MET1 – ALA-19 of MmpS5). The first peak maps to the insertion region for the coiled-coil domain, while the second peak maps to the MmpS5/L5 interface (Xiong et al., 2025). Structural studies have suggested that the interface region of periplasmic cavity where the coiled-coil domain attaches, the periplasmic domain, and the coiled-coil domain are highly flexible because they participate in substrate recognition and export (Fountain et al., 2025a; Maharjan et al., 2024; Xiong et al., 2025). Therefore, the changes observed in the shape of Peak 1 and Peak 2 of the ligand-bound systems compared to the apo protein can inform about their mechanism of inhibition.

None of the compounds at site 1 significantly impacted the flexibility of residues away from the flexibility hotspots. NOR and VER binding increased the amplitude of Peak 2 and reduced the breadth of Peak 1, implying that their binding destabilized the MmpS5L5 interface and rigidified the periplasmic cavity. 3920 binding decreased the breadth of Peak 1 without impacting the shape of Peak 2, suggesting that its binding only rigidified the periplasmic cavity. Conversely, 406 and 4787 binding increased the amplitude of Peak 1 without substantially impacting the amplitude of Peak 2, suggesting that their binding destabilized the periplasmic cavity residues. Notably, 406 increased the breadth of Peak 1 while 4787 decreased the breadth of Peak 1, indicating broader flexibility of the 406’s periplasmic domain and rigidification of the 4787 one.

Similarly, the PRMSF profiles of NOR and VER at site 4 closely traced that of the apo across most residues, indicating that their binding did not significantly change the protein’s local flexibility. Notably, Peak 2 for NOR was broader than the apo’s, indicating destabilization of the MmpS5L5 interface. Conversely, the PRMSF profiles for all the potential hits had an elevated and broader Peak 1 and elevated Peak 2, indicating that their binding destabilized these regions. Notably, the 10367 complex had a higher PRMSF value for most residues, highlighting potential destabilization of the protein’s local structure. These residue-level patterns are consistent with the broader structural instability observed for 7104 and 10367 in the RMSD analyses, while further supporting the comparatively stable behaviour of 4031.

**Figure 8:**
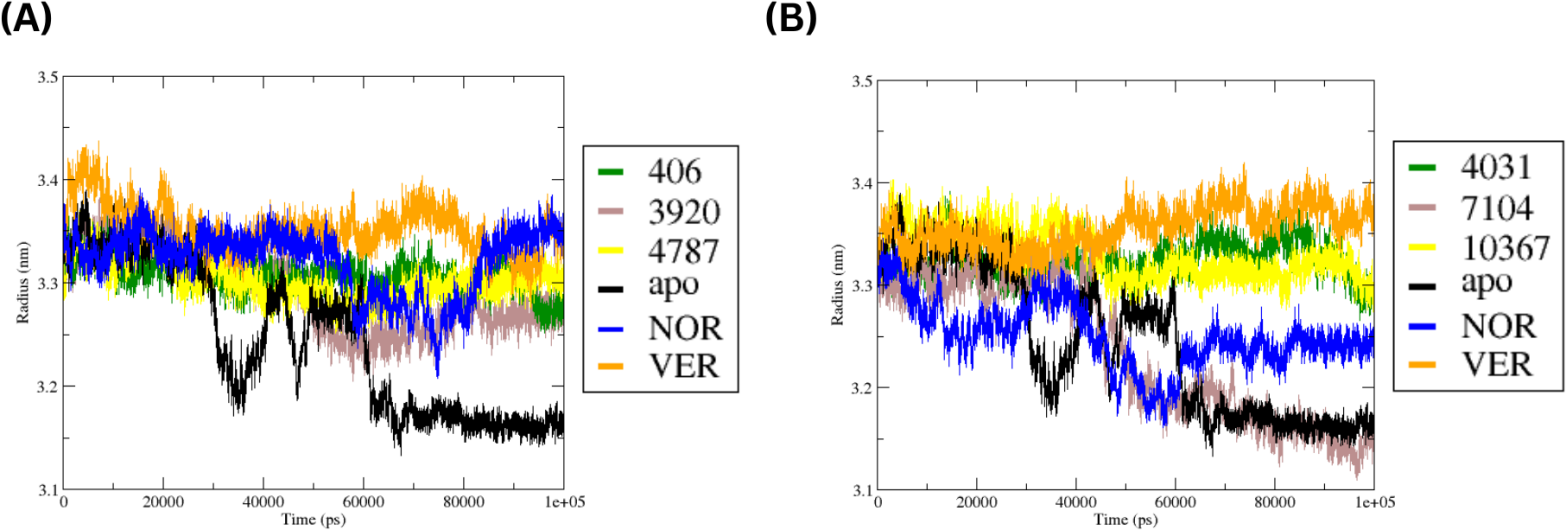
Time-series plots of the Rg values for the MmpS5L5-apo, -inhibitor, and -hit complexes at Site 1 **(A)** and Site 4 **(B)**.

To determine whether ligand binding affected the overall compactness of MmpS5L5, the radius of gyration (Rg) was examined. Briefly, the Rg measures the distribution of atoms around the structure’s centre of mass, with lower values indicating a more compact structure and larger values suggesting structural flexibility. The time series plots of the Rg of the MmpS5L5 apo, -inhibitor and -hit systems at Site 1 and 4 are depicted in **Figures 8A, B** respectively.

The apo system exhibited the largest fluctuations in Rg values, suggesting significant changes in protein compactness during the simulation - consistent with the flexible nature of MmpS5L5. Notably, the apo system had the highest decline in Rg values after 60 ns, supporting progressive structural compaction. In contrast, the site 1 complexes exhibited a narrow Rg range (3.15 – 3.40 nm), implying that ligand binding did not cause any significant unfolding. Across the ligand-bound complexes, VER maintained the highest Rg values across the simulation, suggesting a more expanded conformation and consistent with its higher PRMSD trend. NOR, 406, and 4787 systems showed moderate and stable Rg profiles, matching their moderate PRMSD values. The 3920 complex exhibited slightly lower values after 50 ns, indicating a more compact structure in the later stages of the simulation – like the apo.

The Rg profiles at site 4 indicate that the overall fold was maintained in all systems, but the degree of compactness varied among the complexes. The 4031 system showed the most noticeable decrease in Rg after 50 ns similar to the apo system, suggesting progressive structural compaction and congruent with its low PRMSD. VER exhibited a similar Rg trend, although it later stabilized at slightly higher values than the apo and 4031 complex. In contrast, NOR maintained the highest Rg values for most of the simulation, indicating a less compact structure and supporting moderate structural rearrangement – consistent with its broad, bimodal PRMSD. The 7104 and 10367 complexes maintained an intermediate range with moderate fluctuations – consistent with their moderate, unimodal PRMSD.

**Figure 9:**
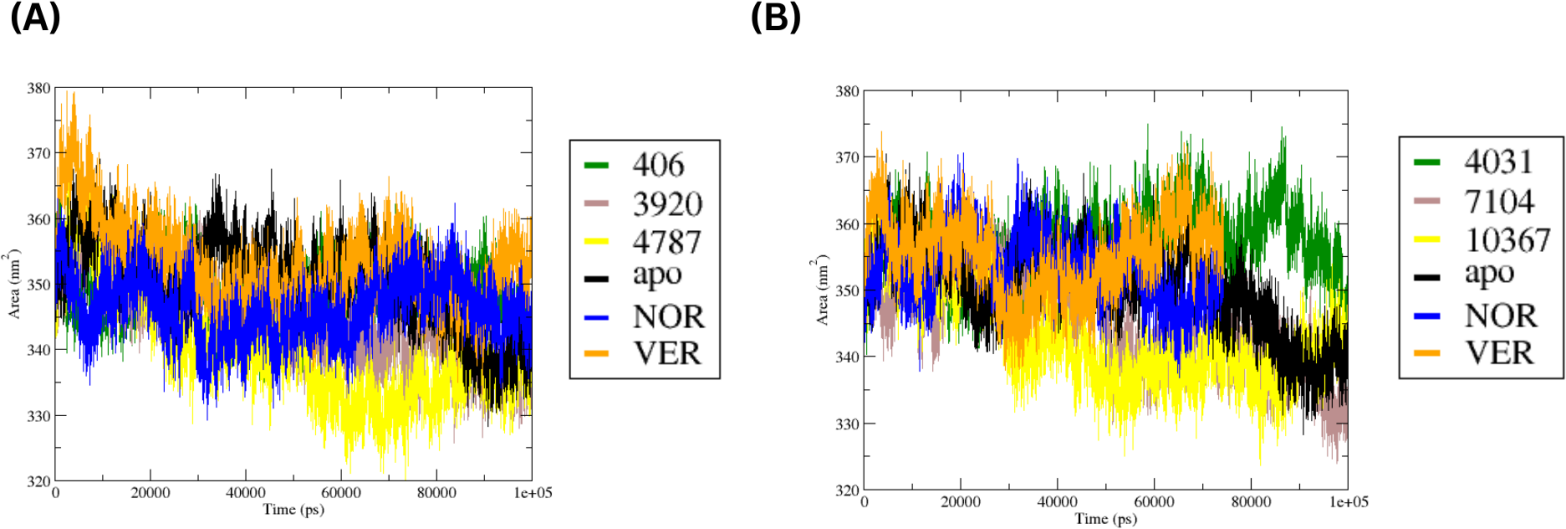
Time-series plots of the SASA values for the MmpS5L5-apo, -inhibitor, and -hit complexes at Site 1 **(A)** and Site 4 **(B)**.

The SASA profiles of the systems were probed to gain further insight into changes in protein folding and solvent exposure during the simulations. Briefly, higher SASA values suggest a more open structure whereas lower values suggest a more compact structure. The SASA profiles of the MmpS5L5 apo, -inhibitor and -hit systems at Site 1 and 4 are depicted in **Figures 9A, B** respectively.

At site 1, all ligand-bound systems remained relatively stable, with only moderate changes in surface exposure over time. The apo system shifted towards lower SASA values after 60 ns, indicating progressive structural compaction – congruent with its Rg trend. A similar trend was observed for the 4787, NOR, and 3920 complexes, indicating tighter packing over time. Conversely, VER maintained higher SASA values throughout the simulation, suggesting it maintained a more open conformation and supporting its higher PRMSD and Rg values. A similar trend was observed for the 406 complex – aligning with its moderate PRMSD and Rg values. Taken together, the RMSD, PRMSF, Rg, and SASA analyses consistently identify 3920 as the most structurally stable hit at site 1.

Similarly, the SASA plots show that ligand binding at site 4 did not substantially alter the overall protein structure as only moderate variations in SASA values were observed over time. The 4031 complex displayed the greatest reduction in SASA values as the simulation progressed akin to the apo system, highlighting increased protein compaction. 7104 exhibited a similar trend, but the decrease was less pronounced, congruent with stabilization of the protein’s structure. Conversely, the 10367 system maintained higher SASA values, matching its high PRMSD and Rg values - suggesting global structure destabilization. The SASA values for NOR and VER complexes were in the intermediate range, suggesting some variation but no substantial fluctuation in the surface exposure. Collectively, the RMSD, PRMSF, Rg, and SASA analyses consistently revealed 4031 as the most structurally stable hit at site 4.

### 3.4 Conformational dynamics

**Figure 10:**
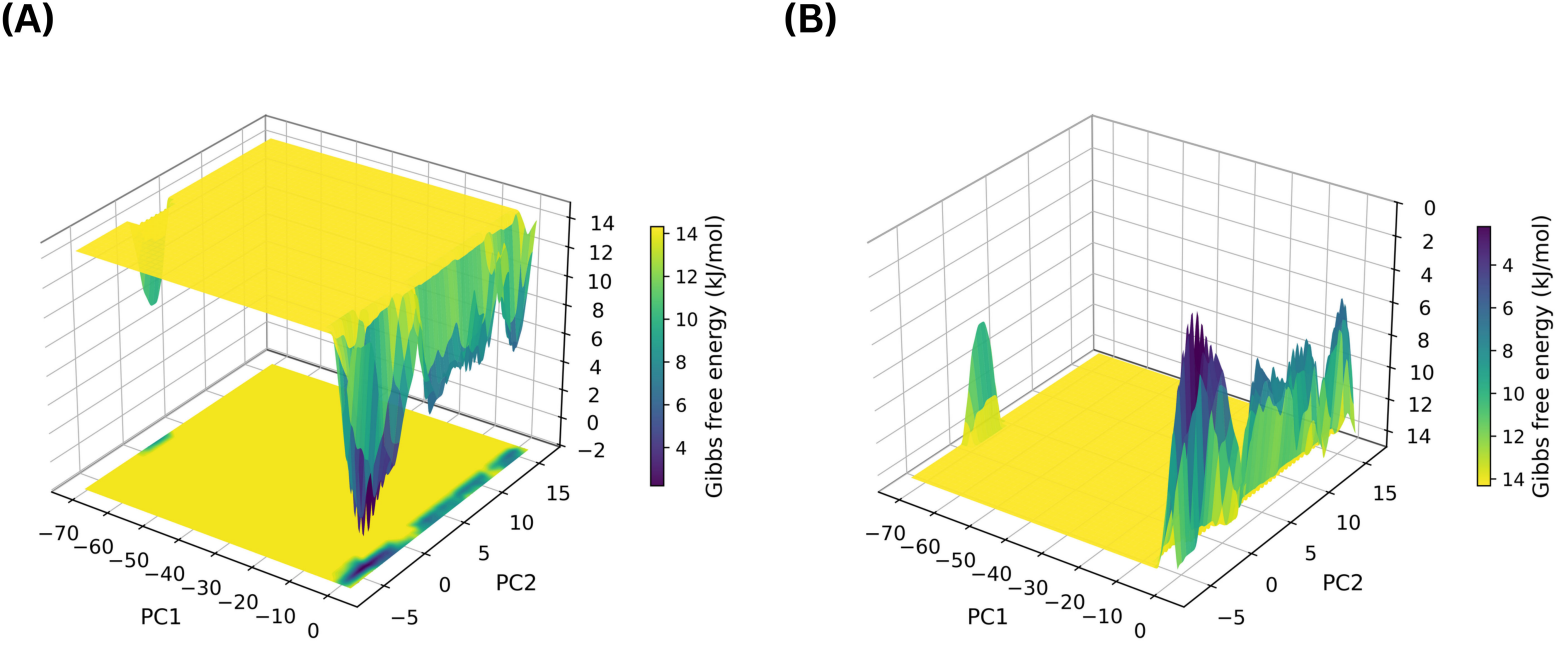
The 3D normal **(A)** and inverted **(B)** PCA-FEL graphical representation of the conformational dynamics of the apo MmpS5L5 protein.

To further assess the impact of ligand binding on MmpS5L5 dynamics, PCA was conducted to assess large-scale conformational motions while analysis of the FEL was performed to evaluate the thermodynamic stability of the different conformations sampled. Whereas the structural stability metrics above describe how stably the complexes behaved over time, PCA–FEL provides complementary insight into whether ligand binding restricts or broadens the conformational space accessible to MmpS5L5. The 3D diagram of the normal and inverted PCA-FEL for apo MmpS5L5 is given in **Figures 10A, B** respectively.

**Figure 11:**
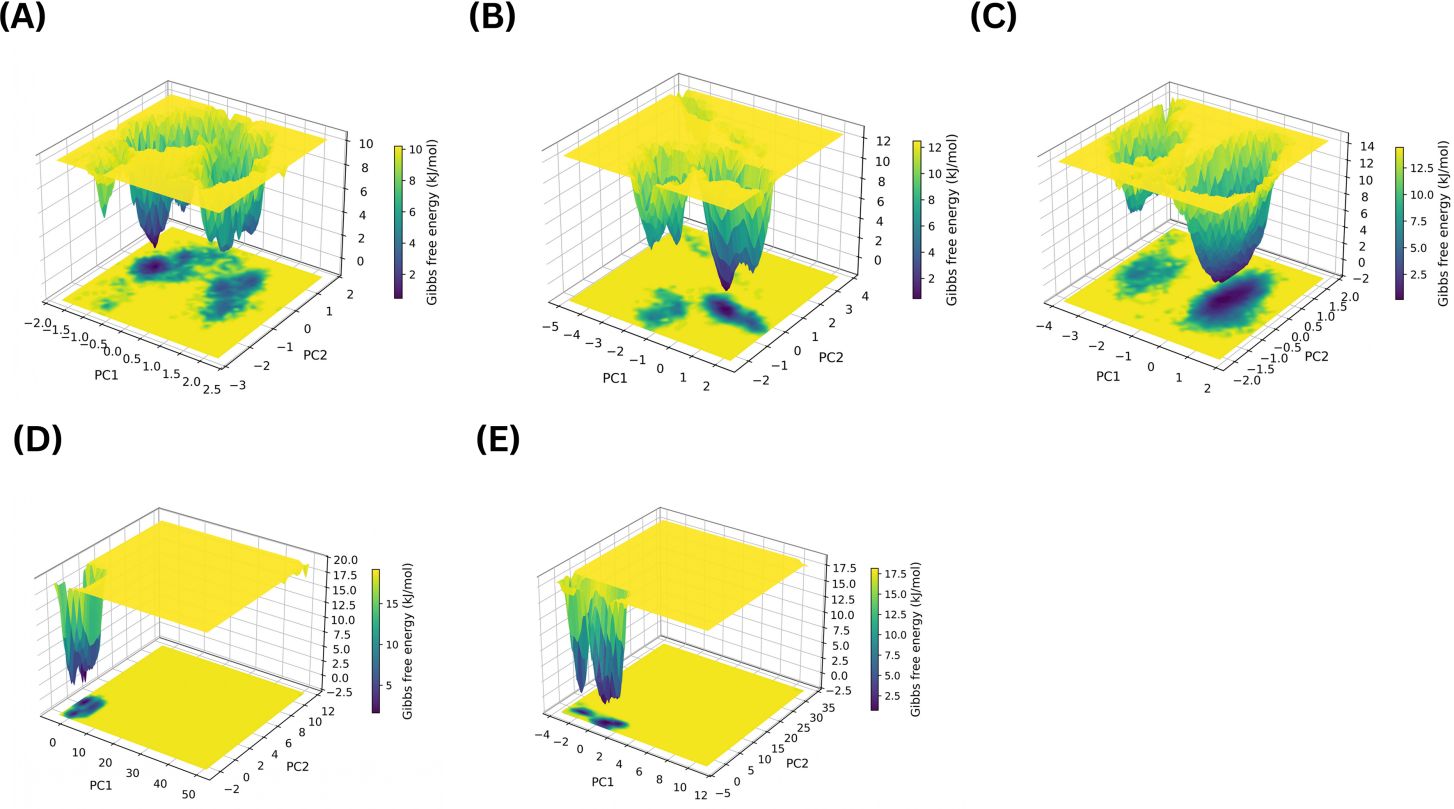
The 3D diagrams of the normal PCA-FEL for MmpS5L5 complexes with NOR **(A)**, VER **(B)**, 406 **(C)**, 3920 **(D)**, and 4787 **(E)** bound in Site 1.

The apo PCA-FEL consisted of a broad accessible landscape with multiple, narrow interconnected basins, indicating that the energy barriers between the different conformational substates sampled were low. This enhanced conformational sampling is typical of apo transporter systems that need conformational flexibility to function, such as MmpS5L5 (Lazarova et al., 2024; Lewis et al., 2023). Although the protein samples multiple conformational substates because of the local flexibility of its gating and substrate binding domains, it does not unfold and its overall structure remains fairly constant, consistent with the observed low PRMSD. Against this baseline, the PCA-FELs of the ligand-bound systems were then examined to determine whether binding promoted conformational trapping or sampling. The 3D diagrams of the normal PCA-FELs for the complexes of NOR, VER, 406, 3920, and 4787 bound at site 1 are provided in **Figure 11**.

The PCA-FELs of the site 1 complexes revealed distinct ligand-dependent effects on conformational sampling. The NOR PCA-FEL diagram, as shown in **Figure 11A**, shows that the complex had multiple shallow, interconnected basins – indicating that it easily transitioned between multiple low-energy conformational substates. This conformational plasticity was consistent with its low PRMSD, high LRMSD, stable interactions with GLN-196, and strong destabilization of its PRMSF Peak 2, pointing to possible efflux inhibition by impairing MmpS5L5’s substrate recognition ability and not conformational trapping. In contrast, VER PCA-FEL depicted in **Figure 11B** consisted of two dominant basins separated by moderate energy barriers, indicating that the system sampled two conformational substates. Taken together with its bimodal PRMSD, low LRMSD, and moderate destabilization of its PRMSF Peak 2, VER PCA-FEL suggests potential efflux inhibition by conformational trapping.

Among the site 1 hits, the PCA-FEL of 3920 shown in **Figure 11C** and 4787 illustrated in **Figure 11E** had a single narrow, deep basin, indicating that ligand binding restricted MmpS5L5 conformational ensemble. Markedly, the 3920 PCA-FEL was more constricted than the 4787 one, in line with the 3920 complex displaying a lower PRMSD, LRMSD, and narrower PRMSF Peak 2. Conversely, the 406 PCA-FEL depicted in **Figure 11D** consisted of two dominant broad basins separated by moderate energy barriers, indicating greater conformational exploration and consistent with its bimodal PRMSD, high LRMSD, and increased breadth and height of its PRMSF Peak 1. These results further support 3920 as the most promising site 1 hit.

**Figure 12:**
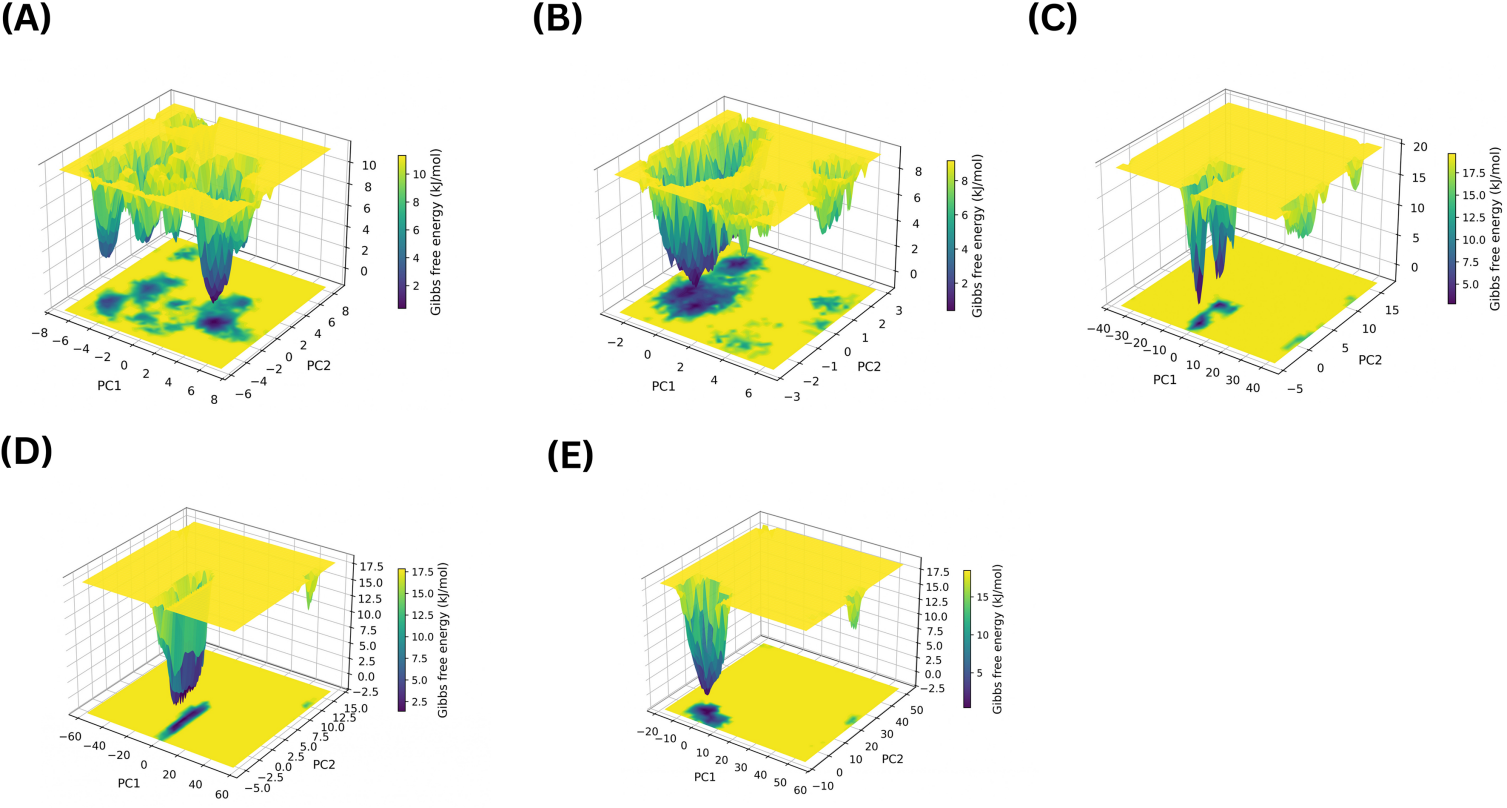
The 3D diagrams of the normal PCA-FEL for MmpS5L5 complexes with NOR **(A)**, VER **(B)**, 4031 **(C)**, 7104 **(D)**, and 10367 **(E)** bound in Site 4.

Similarly, the PCA-FELs of the site 4 complexes pointed to differential effects on protein dynamics. The 3D diagrams of the normal PCA-FELs for the complexes of NOR, VER, 406, 3920, and 4787 bound at site 4 are illustrated in **Figure 12**.

NOR’s PCA-FEL illustrated in **Figure 12A** had multiple narrow basins, suggesting that the complex sampled multiple conformational substates. Most of the basins being interconnected implies that the transitions between these states were energetically favourable – consistent with increased conformational flexibility. This enhanced conformational sampling could be due to NOR binding altering the coupling of MmpS5 to MmpL5, as indicated by its broadened PRMSF Peak 2 and bimodal PMRSD. In contrast, VER’s PCA-FEL displayed in **Figure 12B** consisted of one broad, deep basin, indicating that ligand-protein complex sampled a single stable conformational ensemble. This highly stable conformation was corroborated with VER forming persistent contacts with both MmpL5 and MmpS5 residues, leading to a lower PRMSD, LRMSD, and apo-like PRMSF profile.

Conversely, all three site 4 hits constricted MmpS5L5’s conformational sampling but to varying extents, implying potential efflux inhibition through conformational trapping. Among them, 4031 PCA-FEL shown in **Figure 12C** occupied a more confined conformational space, matching its lower PRMSD and LRMSD. As illustrated in **Figure 12E**, 10367 complex displayed the least confined conformational ensemble along PC1, consistent with its higher PRMSD, LRMSD, and higher PRMSF values for most residues. 7104, as shown in **Figure 12E**, explored a broader conformational space than 4031, in line with its elevated PRMSD, LRMSD, and greater destabilization of its PRMSF Peak 1 and 2. Together, these findings reinforce 4031 as the most promising site 4 hit.

### 3.5 Binding free energy and hit selection

To complement the structural and conformational analyses, MMGBSA calculations were used to estimate the binding free energies of the reference inhibitors and the six selected hits over the full 100 ns MD trajectories. MMGBSA analysis combines entropy, solvent solvation effects, and molecular mechanics to calculate the free energy of ligand binding to target proteins. As such, it gives a better estimation of thermodynamic stability and strength of the interactions between ligands and proteins, successfully reproducing experimental inhibitory activity (Genheden and Ryde, 2015). Consequently, the MMGBSA results were used to rank the hits and identify the most promising ones.

The MMGBSA free energies for the inhibitors and hits at site 1 of MmpS5L5 are given in **Table 2**.

**Table 2:** MMGBSA free energies for the known inhibitors and hits 406, 3920, and 4787.

Complex	Total ΔG bind	Coul	Cov	H-bond	Lipo	Solv_GB	VdW	Ligand efficiency (ln)
NOR	-108.37± 8.44	46.66 ± 58.99	5.47 ± 8.01	-1.64 ± 3.59	-39.88 ± 3.42	-18.26 ± 3.22	-98.06 ± 7.11	-15.16 ± 3.98
VER	-103.32 ± 12.74	25.88 ± 65.89	2.17 ± 5.32	-0.56 ± 1.28	-21.89 ± 5.15	1.96 ± 35.890	-92.64 ± 11.27	-14.47± 6.35
406	-92. 86 ± 15.78	97.76 ± 44.03	5.65 ± 4.71	0.24 ± 2.67	-14.78 ± 5.32	-49.08 ± 38.45	-74.89 ± 13.08	-12.08 ± 3.63
3920	-111.81 ± 8.98	43.77 ± 57.89	3.14 ± 5.90	-2.04 ± 1.78	-44.56 ± 5.67	-26.78 ± 61.08	-89.57 ± 8.09	-16.46 ± 4.06
4787	-96. 00 ± 13.47	92.95 ± 67.87	4.23 ± 4.21	1.326 ± 4.63	-19.35 ± 7.56	-54.30 ± 64.43	-88.07± 10.83	-13.57 ± 2.99

Coul, Coulombic energy; Cov, Covalent binding energy; VdW, Van der Waals energy; Lipo, Lipophilic energy; Solv_GB, Generalized Born electrostatic solvation energy; H-bond, Hydrogen-bonding energy. All energies are in kcal/mol.

Across all the ligand-protein systems, the total ΔG bind was largely driven by Van der Waals and lipophilic energies, consistent with stable packing within a hydrophobic cavity like site 1. VER and NOR exhibited strong stabilization of MmpS5L5, with their total ΔG bind being -103.32 and -108.37 kcal/mol respectively. Notably, 3920 displayed the most stable binding energetics among the ligands because it had the lowest ligand efficiency (-16.46 ± 4.06 kcal/mol) and total average ΔG bind (-111.81 ± 8.98 kcal/mol). By contrast, 406 and 4787 had weaker total binding free energies of -92.86 ± 15.78 and -96.00 ± 13.47 kcal/mol, respectively. The favorable energetics for 3920 complex reinforce the structural and dynamic stability trends observed in its PRMSD, LRMSD, PRMSF, Rg, SASA, and PCA-FEL analyses.

The MMGBSA free energies for the inhibitors and hits at site 4 of MmpS5L5 are given in **Table 3**.

**Table 3:** MMGBSA free energies for the inhibitors and hits 4031, 7104, and 10367.

Complex	Total ΔG bind	Coul	Cov	H-bond	Lipo	Solv_GB	VdW	Ligand efficiency (ln)
NOR	-101.11 ± 7.04	66.53 ± 51.87	1.95 ± 3.26	-1.24 ± 0.88	-39.83 ± 2.03	-34.88 ± 51.07	-91.76 ± 5.52	-15.35 ± 1.07
VER	-108.70 ± 9.69	62.33 ± 33.77	4.99 ± 3.85	-1.23 ± 0.66	-43.33 ± 2.10	-107.08 ± 15.21	-71.12 ± 5.93	-16.50 ± 1.71
4031	-109.56 ± 8.40	94.87 ± 48.51	3.88 ± 3.65	-2.02 ± 0.87	-44.25 ± 2.70	-58.39 ± 45.72	-102.67 ± 6.13	-17.63 ± 1.27
7104	-97.43 ± 9.74	80.95 ± 50.50	4.29 ± 2.54	-1.09 ± 0.92	-37.27 ± 2.84	-49.54 ± 48.84	-92.19 ± 8.82	-14.91 ± 1.48
10367	-91.11 ± 9.41	101.88 ± 58.50	4.49 ± 3.67	-0.79 ± 0.97	-36.09 ± 2.94	-70.93 ± 54.83	-87.00 ± 6.87	-13.84 ± 1.43

Coul, Coulombic energy; Cov, Covalent binding energy; VdW, Van der Waals energy; Lipo, Lipophilic energy; Solv_GB, Generalized Born electrostatic solvation energy; H-bond, Hydrogen-bonding energy. All energies are in kcal/mol.

Like site 1, the total average ΔG bind across all systems for site 4 was largely driven by Van der Waals and lipophilic energies, supporting the importance of hydrophobic interactions between MmpS5 and MmpL5 amino acid residues at the interface in stabilizing the complex. VER and NOR exhibited strong stabilization of MmpS5L5, with their total ΔG bind being -108.70 and -101.11 kcal/mol. 4031 displayed the most stable binding energetics among the hits because it had the lowest average ligand efficiency (-17.63 ± 1.27 kcal/mol) and total ΔG bind (-109.56 ± 8.40 kcal/mol), matching its low LRMSD and PRMSD, stable ligand-protein contacts, and constrained PCA-FEL profile. 10367 has the least stable binding energetics since it had the highest average ligand efficiency and total ΔG bind, consistent with its high PRMSD, LRMSD, SASA, and Rg values.

Taken together, the MMGBSA results validate the trends observed in the docking, MD stability, and PCA–FEL analyses. Compound 3920 emerged as the strongest site 1 hit because it combined favourable residue contacts, stable binding behaviour, restricted conformational sampling, and the most favourable average binding free energy. Likewise, 4031 was identified as the most promising site 4 hit on the basis of its stable structural profile, constrained FEL, and highly favourable energetic profile. Overall, these findings support prioritization of 3920 and 4031 as the most promising ReFRAME-derived scaffolds for further empirical evaluation as putative MmpS5L5 inhibitors.

## 4 Conclusion

This study provides a comprehensive *in silico* framework for the unveiling of potential inhibitors of *Mtb* MmpS5L5’s efflux function from the largest drug repurposing library. Of the ReFRAME clinical-stage drugs screened, 3920 and 4031 were revealed as the most promising hits because of their favourable structural, thermodynamic and conformational dynamics. Notably, PCA-FEL and MMGBSA analyses demonstrated that the predicted binding affinities of the two compounds onto MmpS5L5 was stable, energetically favourable and constrained in its scope of conformational sampling, highlighting its potential to inhibit MmpS5L5 function through sustained conformational trapping. Despite these promising *in silico* results, the data warrants additional experimental validation through mutational studies, protein and cellular level *in vitro* assays, and experimental structural characterisation of MmpS5L5 complexed with 3920 and 4031 as the computational results are just predictions and may not translate to empirical inhibition of MmpS5L5. Moreover, structure-guided efforts to optimise the identified scaffolds to enhance selectivity and potency are also recommended. Altogether, our study has uncovered putative, viable *Mtb* mmpS5L5 targeting drug candidates.

## Data Availability

The original contributions presented in the study are included in the article/supplementary material. Further inquiries can be directed to the corresponding author.

## References

[B1] Abraham M. J. Murtola T. Schulz R. Páll S. Smith J. C. Hess B. . (2015). GROMACS: high performance molecular simulations through multi-level parallelism from laptops to supercomputers. Softwarex 1–2, 19–25. doi: 10.1016/j.softx.2015.06.001. PMID: 41940325

[B2] Amadei A. Linssen A. B. Berendsen H. J. (1993). Essential dynamics of proteins. Proteins 17, 412–425. doi: 10.1002/prot.340170408. PMID: 8108382

[B3] Baltrukevich H. Podlewska S. (2022). From data to knowledge: systematic review of tools for automatic analysis of molecular dynamics output. Front. Pharmacol. 13. doi: 10.3389/fphar.2022.844293. PMID: 35359865 PMC8960308

[B4] Bharadwaj K. K. Ahmad I. Pati S. Ghosh A. Rabha B. Sarkar T. . (2024). Screening of phytocompounds for identification of prospective histone deacetylase 1 (HDAC1) inhibitor: an in silico molecular docking, molecular dynamics simulation, and MM-GBSA approach. Appl. Biochem. Biotechnol. 196, 3747–3764. doi: 10.1007/s12010-023-04731-3. PMID: 37776441

[B5] Biswas S. S. Browne R. B. Borah V. V. Roy J. D. (2021). In silico approach for phytocompound-based drug designing to fight efflux pump-mediated multidrug-resistant Mycobacterium tuberculosis. Appl. Biochem. Biotechnol. 193, 1757–1779. doi: 10.1007/s12010-021-03557-1. PMID: 33826064 PMC8024441

[B6] Breznik M. Ge Y. Bluck J. P. Briem H. Hahn D. F. Christ C. D. . (2023). Prioritizing small sets of molecules for synthesis through in‐silico tools: a comparison of common ranking methods. ChemMedChem 18, e202200425. doi: 10.1002/cmdc.202200425. PMID: 36240514 PMC9868080

[B7] Bruch E. M. Petrella S. Bellinzoni M. (2020). Structure-based drug design for tuberculosis: challenges still ahead. Appl. Sci. 10, 4248. doi: 10.3390/app10124248. PMID: 41725453

[B8] Chacha R. Maina C. Murungi E. Guantai E. Ngugi M. Ndirangu E. . (2025). Potentiation of the antimycobacterial activity of bedaquiline, clofazimine, and doxycycline against Mycobacterium smegmatis by several natural product-based compounds is putatively via efflux inhibition. Open Res Africa. 8 (23), 1–30. doi: 10.12688/openresafrica.16071.1 PMC13077286 41988094

[B9] Ejalonibu M. A. Ogundare S. A. Elrashedy A. A. Ejalonibu M. A. Lawal M. M. Mhlongo N. N. . (2021). Drug discovery for Mycobacterium tuberculosis using structure-based computer-aided drug design approach. Int. J. Mol. Sci. 22, 13259. doi: 10.3390/ijms222413259. PMID: 34948055 PMC8703488

[B10] Farhadi B. Beygisangchin M. Ghamari N. Jakmunee J. Tang T. (2025). Molecular dynamics simulations of proteins: an in-depth review of computational strategies, structural insights, and their role in medicinal chemistry and drug development. Biol. Cybern. 119, 28. doi: 10.1007/s00422-025-01026-0. PMID: 41003729

[B11] Fountain A. Waller N. J. E. Cheung C.-Y. Jowsey W. Chrisp M. T. Troll M. . (2025a). Verapamil and its metabolite norverapamil inhibit the Mycobacterium tuberculosis MmpS5L5 efflux pump to increase bedaquiline activity. Proc. Natl. Acad. Sci. U.S.A. 122, e2426827122. doi: 10.1073/pnas.2426827122. PMID: 40244664 PMC12036985

[B12] Fountain J. Böhning J. McLaughlin S. H. Morgan T. E. Edelstein P. H. Troll M. . (2025b). Structural and functional analysis of the Mycobacterium tuberculosis MmpS5L5 efflux pump presages increased bedaquiline resistance. Proc. Natl. Acad. Sci. U.S.A. 122, e2516660122. doi: 10.1073/pnas.2516660122. PMID: 40986343 PMC12501195

[B13] Friesner R. A. Murphy R. B. Repasky M. P. Frye L. L. Greenwood J. R. Halgren T. A. . (2006). Extra precision Glide: docking and scoring incorporating a model of hydrophobic enclosure for protein-ligand complexes. J. Med. Chem. 49, 6177–6196. doi: 10.1021/jm051256o. PMID: 17034125

[B14] Ge Y. Pande V. Seierstad M. J. Damm-Ganamet K. L. (2024). Exploring the application of SiteMap and Site Finder for focused cryptic pocket identification. J. Phys. Chem. B 128, 6233–6245. doi: 10.1021/acs.jpcb.4c00664. PMID: 38904218

[B15] Genheden S. Ryde U. (2015). The MM/PBSA and MM/GBSA methods to estimate ligand-binding affinities. Expert Opin. Drug Discov. 10, 449–461. doi: 10.1517/17460441.2015.1032936. PMID: 25835573 PMC4487606

[B16] Ghajavand H. Kargarpour Kamakoli M. Khanipour S. Pourazar Dizaji S. Masoumi M. Rahimi Jamnani F. . (2019). Scrutinizing the drug resistance mechanism of multi- and extensively-drug resistant Mycobacterium tuberculosis: mutations versus efflux pumps. Antimicrob. Resist. Infect. Control 8, 70. doi: 10.1186/s13756-019-0516-4. PMID: 31073401 PMC6498538

[B17] Graciaa D. S. Schechter M. C. Fetalvero K. B. Cranmer L. M. Kempker R. R. Castro K. G. (2023). Updated considerations in the diagnosis and management of tuberculosis infection and disease: integrating the latest evidence-based strategies. Expert Rev. Anti Infect. Ther. 21, 595–616. doi: 10.1080/14787210.2023.2207820. PMID: 37128947 PMC10227769

[B18] Hu X. Wu Z. Lei J. Zhu Y. Gao J. (2025). Prevalence of bedaquiline resistance in patients with drug-resistant tuberculosis: a systematic review and meta-analysis. BMC Infect. Dis. 25, 689. doi: 10.1186/s12879-025-11067-2. PMID: 40355818 PMC12067902

[B19] Janes J. Young M. E. Chen E. Rogers N. H. Burgstaller-Muehlbacher S. Hughes L. D. . (2018). The ReFRAME library as a comprehensive drug repurposing library and its application to the treatment of cryptosporidiosis. Proc. Natl. Acad. Sci. U.S.A. 115, 10750–10755. doi: 10.1073/pnas.1810137115. PMID: 30282735 PMC6196526

[B20] Karelina M. Noh J. J. Dror R. O. (2023). How accurately can one predict drug binding modes using AlphaFold models? eLife 12, RP89386. doi: 10.7554/eLife.89386. PMID: 38131311 PMC10746139

[B21] Kingdon A. Alderwick L. J. (2021). Structure-based in silico approaches for drug discovery against Mycobacterium tuberculosis. Comput. Struct. Biotechnol. J. 19, 3708–3719. doi: 10.1016/j.csbj.2021.06.034. PMID: 34285773 PMC8258792

[B22] Kordylewski S. K. Bugno R. Podlewska S. (2025). Residence time in drug discovery: current insights and future perspectives. Pharmacol. Rep. 77, 851–873. doi: 10.1007/s43440-025-00748-z. PMID: 40489055 PMC12241192

[B23] Kurki M. Nesterenko A. M. Alsaker N. E. M Ferreira T. Kyllönen S. Poso A. . (2024). Solid-state NMR validation of OPLS4: structure of PC-lipid bilayers and its modulation by dehydration. J. Phys. Chem. B 128, 12483–12492. doi: 10.1021/acs.jpcb.4c04719. PMID: 39651780 PMC11664586

[B24] Laws M. Jin P. Rahman K. M. (2022). Efflux pumps in Mycobacterium tuberculosis and their inhibition to tackle antimicrobial resistance. Trends Microbiol. 30, 57–68. doi: 10.1016/j.tim.2021.05.001. PMID: 34052094

[B25] Lazarova M. Eicher T. Börnsen C. Zeng H. Athar M. Okada U. . (2024). Conformational plasticity across phylogenetic clusters of RND multidrug efflux pumps and its impact on substrate specificity. bioRxiv. doi: 10.1101/2024.11.22.624703. PMID: 41298458 PMC12749989

[B26] Lewis B. Uddin M. R. Moniruzzaman M. Kuo K. M. Higgins A. J. Shah L. M. N. . (2023). Conformational restriction shapes the inhibition of a multidrug efflux adaptor protein. Nat. Commun. 14, 3900. doi: 10.1038/s41467-023-39615-x. PMID: 37463890 PMC10354078

[B27] Madadi-Goli N. Ahmadi K. Kamakoli M. K. Azizi M. Khanipour S. Dizaji S. P. . (2024). The importance of heteroresistance and efflux pumps in bedaquiline-resistant Mycobacterium tuberculosis isolates from Iran. Ann. Clin. Microbiol. Antimicrob. 23, 36. doi: 10.1186/s12941-024-00694-3. PMID: 38664815 PMC11046812

[B28] Maharjan R. Zhang Z. Klenotic P. A. Gregor W. D. Tringides M. L. Cui M. . (2024). Structures of the mycobacterial MmpL4 and MmpL5 transporters provide insights into their role in siderophore export and iron acquisition. PLoS Biol. 22, e3002874. doi: 10.1371/journal.pbio.3002874. PMID: 39423221 PMC11524445

[B29] Manandhar S. Sankhe R. Priya K. Hari G. Kumar B. H. Mehta C. H. . (2022). Molecular dynamics and structure-based virtual screening and identification of natural compounds as Wnt signaling modulators: possible therapeutics for Alzheimer’s disease. Mol. Divers. 26, 2793–2811. doi: 10.1007/s11030-022-10395-8. PMID: 35146638 PMC9532339

[B30] Moussa N. Hassan A. Gharaghani S. (2021). Pharmacophore model, docking, QSAR, and molecular dynamics simulation studies of substituted cyclic imides and herbal medicines as COX-2 inhibitors. Heliyon 7, e06605. doi: 10.1016/j.heliyon.2021.e06605. PMID: 33889764 PMC8047494

[B31] Nada H. Elkamhawy A. Lee K. (2022). Identification of 1H-purine-2,6-dione derivative as a potential SARS-CoV-2 main protease inhibitor: molecular docking, dynamic simulations, and energy calculations. PeerJ 10, e14120. doi: 10.7717/peerj.14120. PMID: 36225900 PMC9549888

[B32] Reading E. Ahdash Z. Fais C. Ricci V. Wang-Kan X. Grimsey E. . (2020). Perturbed structural dynamics underlie inhibition and altered efflux of the multidrug resistance pump AcrB. Nat. Commun. 11, 5565. doi: 10.1038/s41467-020-19397-2. PMID: 33149158 PMC7642415

[B33] Rodrigues L. Cravo P. Viveiros M. (2020). Efflux pump inhibitors as a promising adjunct therapy against drug resistant tuberculosis: a new strategy to revisit mycobacterial targets and repurpose old drugs. Expert Rev. Anti Infect. Ther. 18, 741–757. doi: 10.1080/14787210.2020.1760845. PMID: 32434397

[B34] Saukkonen J. J. Duarte R. Munsiff S. S. Winston C. A. Mammen M. J. Abubakar I. . (2025). Updates on the treatment of drug-susceptible and drug-resistant tuberculosis: an official ATS/CDC/ERS/IDSA clinical practice guideline. Am. J. Respir. Crit. Care Med. 211, 15–33. doi: 10.1164/rccm.202410-2096ST. PMID: 40693952 PMC11755361

[B35] Sethi G. Sahoo S. Han S.-C. Shin D. Hwang J. H. (2026). Computational identification of natural inhibitors targeting GroEL in Leptospira interrogans: an integrative virtual screening and molecular dynamics approach. Front. Cell. Infect. Microbiol. 15. doi: 10.3389/fcimb.2025.1733096. PMID: 41704643 PMC12907346

[B36] Shahbaaz M. Maslov D. A. Vatlin A. A. Danilenko V. N. Grishina M. Christoffels A. (2022). Repurposing based identification of novel inhibitors against MmpS5-MmpL5 efflux pump of Mycobacterium smegmatis: a combined in silico and *In vitro* study. Biomedicines 10, 333. doi: 10.3390/biomedicines10020333. PMID: 35203542 PMC8869396

[B37] Shirts M. R. Klein C. Swails J. M. Yin J. Gilson M. K. Mobley D. L. . (2017). Lessons learned from comparing molecular dynamics engines on the SAMPL5 dataset. J. Comput.-Aided Mol. Des. 31, 147–161. doi: 10.1007/s10822-016-9977-1. PMID: 27787702 PMC5581938

[B38] The UniProt Consortium (2025). UniProt: the universal protein knowledgebase in 2025. Nucleic Acids Res. 53, D609–D617. doi: 10.1093/nar/gkae1010. PMID: 39552041 PMC11701636

[B39] World Health Organization (2025). Global traditional medicine strategy 2025-2034. 1–22. 10.2471/BLT.25.293414 PMC12578523 41180270

[B40] Xiong Z. Yang X. Wang S. Smart C. J. Sisson H. M. Lin Z. . (2025). Structure and assembly of the MmpL5/MmpS5 efflux transporter from Mycobacterium tuberculosis. Nat. Commun. 16, 4976. doi: 10.1038/s41467-025-60365-5. PMID: 40442140 PMC12122801

[B41] Xu J. Tasneen R. Peloquin C. A. Almeida D. V. Li S.-Y. Barnes-Boyle K. . (2017). Verapamil increases the bioavailability and efficacy of bedaquiline but not clofazimine in a murine model of tuberculosis. Antimicrob. Agents Chemother. 62, e01692-17. doi: 10.1128/AAC.01692-17. PMID: 29038265 PMC5740328

[B42] Yamamoto K. Nakata N. Mukai T. Kawagishi I. Ato M. (2021). Coexpression of MmpS5 and MmpL5 contributes to both efflux transporter MmpL5 trimerization and drug resistance in Mycobacterium tuberculosis. mSphere 6, e00518-20. doi: 10.1128/mSphere.00518-20. PMID: 33408221 PMC7845600

[B43] Zhang B. Li J. Yang X. Wu L. Zhang J. Yang Y. . (2019). Crystal structures of membrane transporter MmpL3, an anti-TB drug target. Cell. 176, 636–648.e13. doi: 10.1016/j.cell.2019.01.003. PMID: 30682372

